# Wheelchair use confidence scale for Arab pediatric manual wheelchair users: preliminary evaluation of its measurement properties

**DOI:** 10.3389/fped.2025.1522475

**Published:** 2025-02-27

**Authors:** Hassan Izzeddin Sarsak, Paula W. Rushton

**Affiliations:** ^1^ Occupational Therapy Program, Batterjee Medical College, Jeddah, Saudi Arabia; ^2^ School of Occupational Therapy, Faculty of Health, Dalhousie University, Nova Scotia, NS, Canada

**Keywords:** children, confidence, manual wheelchair user, self-report, wheelchair service provision

## Abstract

**Introduction:**

This study translated the pediatric Wheelchair Use Confidence Scale for Manual Wheelchair Users (WheelCon-M-P) into Arabic (WheelCon-M-A-P) and evaluated whether the translation produced scores similar to the original English version.

**Methods:**

The English version was first translated into Arabic and then verified by back translation method by expert committee in the field of rehabilitation and wheelchair service provision. The final versions were administered to assess confidence with manual wheelchair use among children. Each participant was asked to complete both the WheelCon-M-P English version and the WheelCon-M-A-P Arabic version in a random sequence. Kappa statistics were used to quantify the level of agreement between scores obtained from both versions.

**Results:**

Participants (*n* = 48) had an average age of 14.2 years, were all bilingual, and 54% were male. Kappa agreement obtained was 0.54 (95% confidence interval, 0.49–0.62) indicating significant moderate agreement between the two versions (*p* < 0.000).

**Discussion:**

This study provides preliminary evidence of a valid WheelCon-M-A-P to assess confidence with manual wheelchair use among Arabic-speaking children. Future studies to further test its psychometric properties are crucial.

## Implications for rehabilitation

• The Arabic pediatric version of the Wheelchair Use Confidence Scale for Manual Wheelchair Users (WheelCon-M-A-P) is now available for Arabic healthcare professionals and can be used with Arabic-speaking pediatric manual wheelchair users in clinical practice and research.
• It is important to assess confidence in wheelchair mobility in pediatric populations. The WheelCon-M-A-P can be used to measure Arabic-speaking wheelchair users' confidence with wheelchair use and provide useful information about a child's belief in his/her ability to perform wheelchair related tasks.
• This study provides an opportunity for identifying areas of low confidence in Arabic- speaking pediatric manual wheelchair users. This will help clinicians make informed decisions when prescribing and training pediatric wheelchair users in wheelchair use and providing confidence-enhancing interventions.

## Introduction

Globally, UNICEF estimated that over 236.4 million (10.1%) children aged 0–17 years have disability (1). The Middle East and North Africa are home to more than 20 million children with disabilities (2, 3). One in seven children in the region has one or more functional difficulties that require the use of assistive technology devices including wheelchairs (4, 5). A properly fitted wheelchair supports both the cognitive and psychosocial development of children with physical disabilities (6, 7). Proper wheelchairs enable children to explore their environments and have increased independence, safety, and quality of life (8–10). They also help promote self-worth, confidence and provide opportunities for social participation (11–14).

Wheelchair confidence, defined as the belief individuals have in their ability to use their wheelchair in a variety of contexts, is a key element in wheelchair provision process (11, 15–23). Manual wheelchair users who demonstrate low confidence with wheelchair use show a low level of participation in activities of daily living (24–26). The prevalence of low wheelchair confidence was determined to be 25%–35% as reported among wheelchair users, hence, wheelchair confidence should be both assessed and considered with interventions of rehabilitation as an influencing factor for self-efficacy and social participation (27). Therefore, self-report outcome measures were developed to assess confidence with wheelchair use. The Wheelchair Use Confidence Scale (WheelCon) was designed to measure confidence with wheelchair use and identify individuals who have low confidence while using wheelchair. There are different WheelCon versions available including the Wheelchair Use Confidence Scale for Manual Pediatric Wheelchair users (WheelCon-M-P). The WheelCon outcome measures have been used in research and clinical settings and proved to be valid and reliable tools (19, 20, 27–30).

There are currently a handful of validated Arabic wheelchair outcome measures and none of them measure confidence with manual wheelchair use (31, 32). Therefore, the purpose of this study was to translate the WheelCon-M-P into Arabic and evaluate its level of kappa agreement with the English version.

## Methods

### Study participants

To be included in this study, children needed to: (1) be between the ages of 5 and 18; (2) use a manual wheelchair daily for a minimum of 6 months prior to the study; and (3) be able to read, write, communicate verbally in Arabic and English, and understand simple instructions. Children with severe cognitive and language disorders or children who had undergone a medical intervention during the previous 6 months that could have affected the study outcomes were excluded. All children who met the inclusion criteria were approached first and educated along with their parents about the study seeking their interest and agreement to participate. All interested children provided assent and informed consent was then obtained from their parents. Children recruited for the purpose of this study using convenience sampling methodology after obtaining permission from their parents.

### Ethical approval

Ethical approval to conduct the current study was first obtained from the clinical research ethical committee of a local school-based rehabilitation center (decision number REVAPP20231103).

### Instrument

The WheelCon-M-P was translated in this study from English to Arabic language. The WheelCon-M-P is a 33-item self-report outcome measurement tool that is used for pediatric manual wheelchair users. It measures wheelchair confidence in the areas of negotiating the physical environment, performing activities in the manual wheelchair, knowledge and problem-solving, advocacy, managing social situations and managing emotions (see Table 1). The response scale is based on a visual 5-level Smiley Face Likert scale, with descriptive anchors for the red face (not confident) and green face (very confident). For scoring the WheelCon-M-P, each response is first transformed to a number with red face = 0 and green face = 4 and then the average questionnaire total score is calculated by adding the scores of each question and dividing it by the number of questions answered. The WheelCon-M-P was developed by adapting the WheelCon-M, French Canadian version based on the perceptions of occupational therapists, parents, and pediatric manual wheelchair users. It is available in both French and English. The French-Canadian version has preliminary evidence of its reliability and validity (33). There is also a Dutch version available for assessing confidence in wheelchair mobility in Dutch youth using a manual wheelchair and it includes items only from the negotiating the environment area (34).

**Table 1:** Focus areas and number of items of the WheelCon-M-P.

Area	# items
Negotiating the physical environment	15
Performing activities in the manual wheelchair	6
Knowledge and problem-solving	4
Advocacy	3
Managing social situations	3
Managing emotions	2
Total	33

### Methodological procedures

The WheelCon-M-P English version developers were approached first to obtain approval to translate it into Arabic and were provided with the final verified Arabic version. The forward-back translation methodology following the World Health Organization (WHO) guidelines and recommended procedures were used in this study (35–37). A committee of three Arab expert professionals in the field of rehabilitation and wheelchair service provision performed the forward translation into Arabic and then it was translated back into English by another three bilingual healthcare professionals who were not familiar with the instrument. The final WheelCon-M-P English version was then compared with the original English version by the primary study researcher to verify quality of translated work and perseverance of intended meaning in all items. Pilot testing was conducted to run cognitive debriefing and test level of understanding of the final Arabic pediatric version of the WheelCon-M-P (WheelCon-M-A-P) by 12 of the study participants. This helped ensure that the Arabic translation is clear, easy to understand, culturally acceptable, and relevant to the original English version.

After approval of the final version of the WheelCon-M-A-P, it was tested in 48 bilingual English-Arabic pediatric manual wheelchair users. All participants were asked to complete both the WheelCon-M-P English version and the WheelCon-M-A-P Arabic version in a random sequence and it took each participant an average of 10–15 min to complete each version. The study principal investigator provided an opportunity for parents to be there while completing the instrument. Also, children felt free to ask questions if they did not understand any of the items in either the English or Arabic version and the interviewer read some of the items for the children once needed.

### Data analyses

Descriptive statistics of simple percentages, means, and standard deviation were used to analyze participants' demographic information. The agreement between the two versions was assessed using kappa coefficient for agreement between the two versions, a 95% confidence interval was constructed for kappa, and the kappa coefficient was interpreted conventionally as follows: values ≤ 0 as indicating no agreement and 0.01–0.20 as none to slight, 0.21–0.40 as fair, 0.41–0.60 as moderate, 0.61–0.80 as substantial, and 0.81–1.00 as almost perfect agreement (38). All statistical analyses were done using IBM-SPSS version 23.0 (39).

## Results

### Demographics of subjects (*n* = 48)

The study sample consisted of 48 pediatric wheelchair users (26 were male and 22 were female). The average child age was 14.2 years old and had used a manual wheelchair for a least the past 6 months (see Table 2).

**Table 2:** Study participants’ demographics (*n* = 48).

Demographics		*n* (%)
Age (mean, SD)	14.2 (±06.45)	
Gender		
Male		26 (54)
Female		22 (46)
Years using wheelchair (mean, SD)	3.8 (±2.48)	
First language		
Arabic		33 (69)
English		15 (31)
Primary diagnosis		
Cerebral palsy		19 (40)
Spina bifida		16 (33)
Muscular dystrophy		13 (27)

### WheelCon-M-P items translation

Per cognitive debriefing and backward translation review, only one minor issue was detected and revealed during the translation in question/item number 12 “I am confident that I can use my manual wheelchair to go push the crosswalk button and cross the street before the traffic light turns red”. In Saudi Arabia and most Arab countries, we don't have crosswalk button next to the traffic light. Therefore, a minor edit was performed while maintaining the original intended meaning and the modified item became “I am confident that I can use my manual wheelchair to go push the crosswalk button and/or cross the street before the traffic light turns red”. The developers were informed of this minor change and provided with the final approved version.

### Kappa measurement of agreement

**Figure 1:**
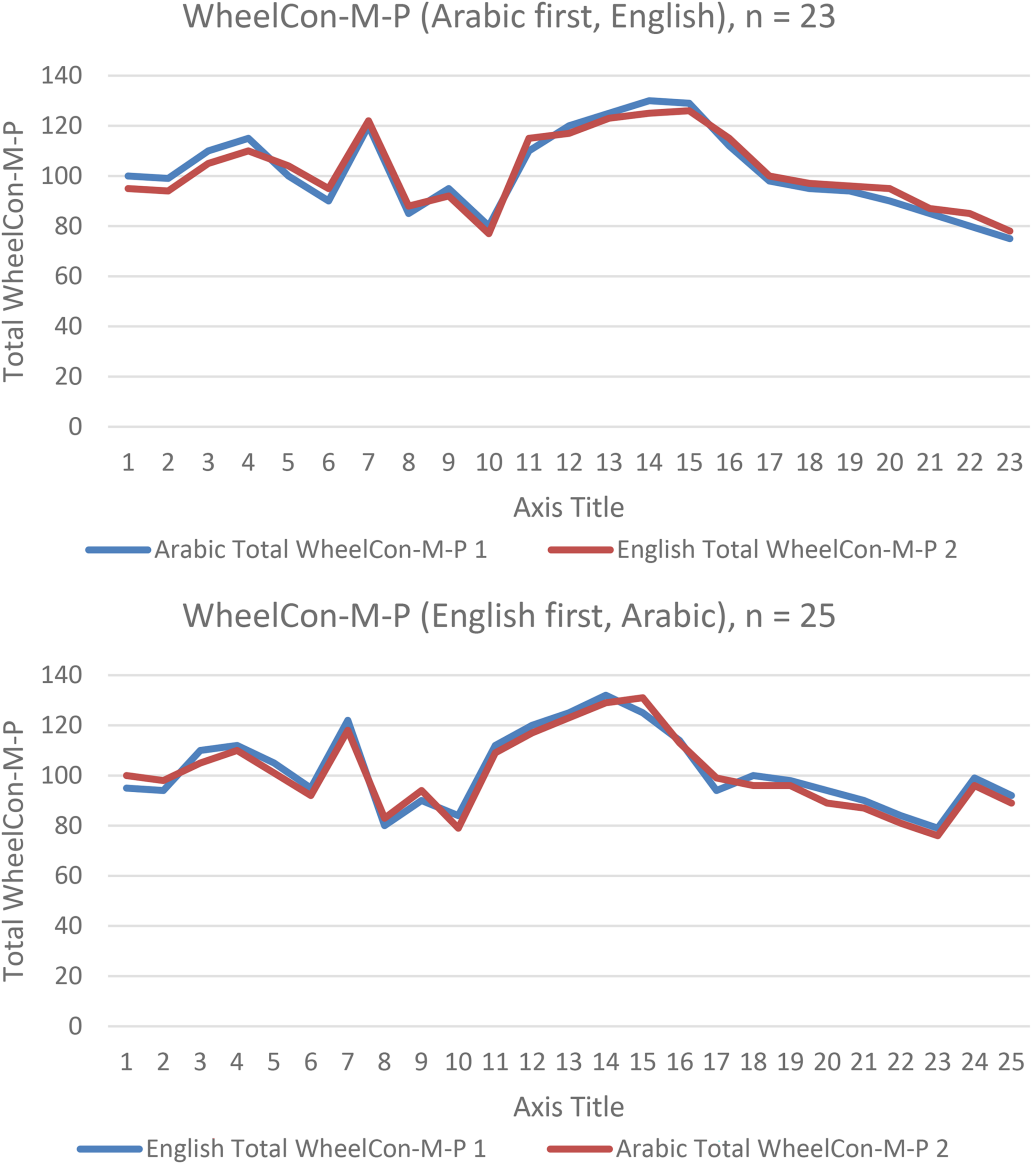
WheelCon-M-P 1 (complete the first WheelCon-M-P), and WheelCon-M-P 2 (complete the second WheelCon-M-P in the other language).

Kappa agreement obtained was 0.54 (95% confidence interval, 0.49–0.62) with percent agreement of over 61% indicating significant moderate agreement between the two versions (*p* < 0.000) (see Figure 1).

## Discussion

The purpose of the current study was to translate the WheelCon-M-P into Arabic and to evaluate its agreement with the original English version. The Wheel-Con-M-P tool measures confidence in wheelchair use for children and has proved to be helpful and useful outcome measure in wheeled mobility and seating assessments. The WheelCon-M, French-Canadian version was adapted into the pediatric version then translated to English and provided a reliable information that helped better understand children confidence while using their wheelchairs (33). The current study is considered the first to translate the WheelCon-M-P tool into Arabic and utilize it with Arabic-speaking wheelchair users' population. In the current study, expert rehabilitation professionals in the field of wheelchair service provision with over 20 years of experience not only performed a rigorous forward and back translation, but a thorough review was conducted to ensure accuracy. In addition, our study reported on the translation of the WheelCon-M-P in which the WHO recommended translation guidelines were followed (35–37). Additionally, our panel of experts ensured maintenance of the original meaning of the items and helped make the Arabic version of the WheelCon-M-P (WheelCon-M-A-P) available for use for Arabic-speaking pediatric wheelchair users and healthcare professionals.

In this study, the participants completed the WheelCon-M-P English and Arabic versions in a random sequence and there were 33 questions with 5 possible answers in the WheelCon-M-P which helped minimize recall, order bias, and improved quality of results. We obtained a significant moderate agreement between English and Arabic WheelCon-M-P versions with highest agreements in items related to negotiating the physical environment, performing activities in the manual wheelchair, knowledge and problem-solving, and advocacy and lower agreements were obtained in items related to managing social situations and managing emotions. This finding was expected and concur with previous studies indicated that social emotional learning process and emotional self-regulation skills in which children learn to better comprehend and manage difficult emotions, express their feelings, develop relationships, and practice social interaction skills especially in critical stressful situations maybe confusing for some children with disabilities and require a lot of training and support to enhance their self-expressive skills and their ability to communicate their emotions with others adequately (40, 41).

To the best of our knowledge, this study was the only study to apply the WheelCon-M-P tool into Arabic context. The statistically significant moderate agreement between English and Arabic WheelCon-M-P versions obtained in the current study is promising and suggests that the translated Arabic version could be helpful and useful to Arabic wheelchair service providers who would like to use it for Arabic-speaking pediatric wheelchair users.

### Limitations of the study

In the current study, participants recruited were from one center only. Future multicenter research studies conducted at more than one medical center or clinic are crucial to better represent the target population. Also, the data derived from this study was self-reported and based on children perceptions only. Further studies that include data derived from focus groups conducted with wheelchair professionals and qualitative interviews conducted with parents of manual wheelchair users are imperative to explore different perspectives on wheelchair confidence and better understand pediatric wheelchair user' needs. For future direction, this would help explore if there are other aspects of wheelchair confidence that are pertinent for Arabic pediatric manual wheelchairs users from the perspectives of children, parents, and healthcare professionals measuring the content validity of the WheelCon-M-A-P (42, 43). Furthermore, this pilot study only examined the level of agreement of the translated version with the original version. Therefore, we are planning to examine other psychometric properties of the WheelCon-M-P Arabic version (WheelCon-M-A-P), such as validity (content and face validity) and reliability (test-retest and inter-rater reliability).

## Conclusion

The WheelCon-M-A-P self-report Arabic tool has been developed for use in research and clinical practice. This study has provided preliminary evidence of new promising, valid, and useful tool for Arab healthcare professionals to help measure confidence with wheelchair use among Arabic-speaking pediatric wheelchair users. Further validation studies are warranted.

## Data Availability

The original contributions presented in the study are included in the article/Supplementary Material, further inquiries can be directed to the corresponding author.
